# Genome Sequence of Resistant *Serratia* sp. Strain HRI, Isolated from a Bottle of Didecyldimethylammonium Chloride-Based Disinfectant

**DOI:** 10.1128/MRA.00095-20

**Published:** 2020-04-30

**Authors:** Samantha J. Mc Carlie, Julius E. Hellmuth, Jeffrey Newman, Charlotte E. Boucher, Robert R. Bragg

**Affiliations:** a Department of Biochemical, Microbial and Food Biotechnology, University of the Free State, Bloemfontein, South Africa; b Lycoming College, Williamsport, Pennsylvania, USA; University of Southern California

## Abstract

Antimicrobial resistance is a significant issue, and it threatens the prevention and effective treatment of a range of bacterial infections. Here, we report the whole-genome sequence of the multidrug-resistant isolate *Serratia* sp. strain HRI. A hybrid assembly was created using sequences from a first (MiSeq) and second (PacBio) sequencing run. This work is imperative for understanding antimicrobial resistance and adds to the knowledge base for combating multidrug-resistant bacteria.

## ANNOUNCEMENT

The emergence of antibiotic resistance and the threat of a postantibiotic era has promoted research into antimicrobial resistance. *Serratia* sp. strain HRI is of interest, as the MICs of this bacterium suggest that it has high disinfectant resistance capabilities.

*Serratia* sp. HRI was first isolated from a bottle of didecyldimethylammonium chloride (DDAC)-based disinfectant (1%) at room temperature at the University of the Free State (UFS) in South Africa.

For both isolation and sequencing procedures, *Serratia* sp. HRI was cultivated on tryptic soy broth agar at 37°C. Total genomic DNA was isolated by manual extraction, as done by Labuschagne and Albertyn (1). The isolate was identified by sequencing the 16S rRNA gene and comparing it to other isolates on the EzBioCloud database (2–5); the closest related type strain was Serratia marcescens subsp. *marcescens* ATCC 13880. The type strain was subsequently obtained from the American Type Culture Collection, and MICs were determined using methods described by Wiegand and coworkers (6) (Table 1).

**TABLE 1:** MICs for *Serratia* sp. strain HRI and the closest related type strain for a variety of quaternary ammonium compound disinfectants

Disinfectant	MIC (μg/ml) of:	
	Serratia marcescens subsp. marcescens ATCC 13880	*Serratia* sp. strain HRI
Virukill	2.8671	43.12494
Benzalkonium chloride	17.227	1,837.5
DDAC	12.79	2,873

Genomic DNA was sent to the UFS Next Generation Sequencing Unit for library preparation and whole-genome sequencing on the Illumina MiSeq platform. An index PCR was done to attach dual indices and Illumina sequencing adapters using the Nextera XT index kit, as per the manufacturer’s instructions. Library concentrations were equalized, pooled, denatured, and transferred to a V3 sequencing cartridge. The MiSeq run proceeded for 600 cycles (2 × 150-bp paired-end reads) and generated 376,021 reads.

A second round of sequencing was conducted at Inqaba Biotechnical Industries (Pretoria, South Africa) to improve sequence quality. Sequencing was done using single-molecule real-time (SMRT) technology using the Sequel PacBio equipment in CCS mode with 50× coverage. Genomic DNA was sheared to ∼10-kb fragments using a Covaris g-TUBE. Thereafter, the sample was end repaired and SMRTbell adaptor ligated. Finally, primer annealing and polymerase binding were completed before sequencing on the PacBio Sequel system, as per the manufacturer’s instructions, generating 180,789 reads.

A hybrid assembly was created using sequences from the MiSeq and PacBio sequencing runs. The *rpoB* gene of *Serratia* sp. HRI was annotated using a previous sequencing run by Rapid Annotations using Subsystems Technology (RAST), and a BLASTn search of this sequence revealed that Serratia marcescens CAV1492 (GenBank accession number CP011642.1) was the closest relative (maximum score, 7,424; total score, 7,424; query cover, 100%; E value, 0.0; percent identity, 99.93%) (7–10). Thus, the genome of CAV1492 was utilized as a reference within the hybrid assembly. Raw reads were quality assessed using FastQC v0.11.8; default parameters were used for all software unless otherwise specified (11), followed by quality filtering using PRINSEQ v0.20.3 (12). Reads obtained were assembled using SPAdes via the hybrid assembly pipeline, and contigs were evaluated using QUAST (13, 14). The assembled genome is 5,885,723 bp long, with a G+C content of 58.4% originating from 105 contigs. This assembly has an *N*_50_ value of 611,273 bp, an *L*_50_ value of 4, and 5,935 coding sequences with 123 RNAs.

### Data availability.

The assembled genome has been deposited into NCBI as BioProject PRJNA580358 and BioSample SAMN13155787, in the Sequence Read Archive under accession numbers SRX7885200 and SRX7109663 for the MiSeq and PacBio data, respectively, and in the DDBJ/ENA/GenBank database under the accession number WIXF00000000. The version described in this paper is the second version, WIXF02000000.
